# Supplementary material for: A Quality of Experience assessment of haptic and augmented reality feedback modalities in a gait analysis system
Source: PLoS One. 2020 Mar 23;15(3):e0230570. doi: 10.1371/journal.pone.0230570 (PMC7089541; doi:10.1371/journal.pone.0230570)
Supplement: Attachment — Submitted filename: Rebuttals_Plos One_TB_Rodrigues.pdf [file pone.0230570.s002.pdf]

A quality of experience assessment of haptic and augmented reality feedback modalities in a gait analysis system

Thiago Braga Rodrigues, Ciarán Ó Catháin, Noel O'Connor, Niall Murray

---

This document includes the reviewers' comments (black text), responses to the reviewer's comments (in blue) and the content which was added to the revised version of the paper (**in blue bold**). We wish to thank all reviewers for this time and efforts to thoroughly review the initial submission. Their constructive comments and critique have greatly improved the version under review now and we genuinely thank them for this.

Reviewer(s)' Comments to Author:

Reviewer #1

In this manuscript, the authors tested effects of haptic and augmented reality feedbacks on varus/valgus motion during walking in healthy young subjects, and evaluated and compared quality of experience between the feedbacks. Please refer to my comments below for improving this manuscript.

Title

Write out QoE. It is very awkward to see an abbreviation in the title: 1) the abbreviation was never introduced in the authors manuscript before, and 2) I am sure there are many potential readers who do not understand what the "QoE" means if they are not written out.

Thanks for the suggestion. The title has been changed to "**A quality of experience assessment of haptic and augmented reality feedback modalities in a gait analysis system**"

Introduction (and Related Work)

We have changed both introduction and Related Work to include reviewer's contribution.

One of the biggest importance of the introduction should be what is the goal of this study, and why is it important to achieve the goal. However, none of these were addressed well in the current introduction. For example, how important giving feedback to patients (e.g., knee oa, arthroplasty) during rehabilitation.

We have added the following excerpt to the introduction:

Feedback is a powerful tool for motor skill learning and helps with the sensory perceptual information as part of performing and learning a skill [6]. The accuracy of exercise performance with feedback in physiotherapy influences the healing process of the patient greatly. Crucial to successful rehabilitation is for the patient to understand the feedback, be it from a clinician or system. Many rehabilitation and retraining systems for physiotherapy and exercise learning use feedback as an important tool for patient learning and progress [7].

Why did authors test haptic and AR feedbacks over other feedbacks?

We have tested haptic because the use of haptic feedback in rehabilitation we deeply explored in many works. We have compared haptic with AR as it was not deeply researched in gait and rehabilitation. The related work section, with new added references gives more explanation about the rationale of using haptic and AR feedback in this work.

...Haptic feedback has been studied in many works related to human activities [17], motor learning [18], and gait retraining [19]. Numerous works have compared haptic feedback with other modalities and have reported haptic: to be "less intrusive" than virtual reality feedback [20]; to be better in supporting task performance when compared to visual feedback for lower extremities [21] in gait; not to affect ecological validity of interaction compared with other modalities [22]; to be easier to understand and followed when compared to auditory and visual stimuli [23]. Haptic feedback was also used to enhance the realism of a walking experience in multimodal environments [24], which could inform the benefit in using it to enhance user experience different applications (e.g. games, movies). Haptic feedback has also been used as an important tool in gait retraining for treatment of knee osteoarthritis [25].

Considering these works, the use of AR for gait feedback has not been deeply explored. There are some exploratory works that suggest employing AR for gait retraining. The results reported in [32] results in significant improvement in gait over a 2D monitor. Other research has reported the use of AR in gait posture training [33] reported statistically significant improvement in posture, balance, and velocity.

Also, why is it important to evaluate quality of experience? At least, these need to be addressed in the introduction.

Despite of the fact that many studies in gait and rehabilitation use objective metrics to assess performance, the perceptual quality of feedback is not deeply explored in this area. With Quality of Experience (QoE), we can evaluate multimedia experiences and feedback in a user centred way that represents the degree of enjoyment or annoyance of a feedback system as in the introduction states. The introduction now clearly addresses this

... . Although these works make a valuable contribution, there was no qualitative metric employed that informs if users were satisfied or enjoyed the feedback experience. This is critical because it informs designers about how the users enjoy, engage, and experience such systems.

Several authors have used QoE assessment in multimedia systems as a paradigm to quantify how various factors of the system influence perceived quality levels from the user perspective. In [35], user QoE levels were compared in an immersive Virtual Reality and Augmented Reality applications. A sample size of twenty-one participants was divided randomly into two groups. Both objective and subjective metrics were gathered. The authors considered system, psychological, and user factor to evaluate quality. The QoE evaluation suggested that users felt safer and accustomed with the use of AR when compared to virtual reality. In [36], a QoE

evaluation of a motor skills rehabilitation game was developed. The authors have assessed QoE through user engagement, task success, interaction, and socialization. This study reported that high QoE scores can be linked to high performance. These works demonstrate the need of a qualitative study for different applications. Several works have found out that valgus/varus incidence is different across gender groups [37, 38]. Anatomical differences between males and females lead to differences in knee alignment. Differences in lower extremity are potential causes of many anterior cruciate ligament in females [39, 40]. Females, in general, have wider hips than males, which is cause of many abnormalities such as knee valgus/varus, with high risk of injuries. Since the incidence of valgus/varus misalignments is different between males and females, we considered gender as an important factor to consider of this study.

For some minor comments, why is “g” always capitalized for “Gait”? Any special reason? I am not sure if Figure 1 is necessary and appropriate. Before the position of Figure 1, AR and haptic was not even introduced. If the authors want to describe what valgus/varus is for readers who are not familiar with the terms, please revise the figure. For the Related Work section, I don’t think this section specifically describes the benefits of haptic and AR feedbacks on valgus/varus during walking. The reference [23], testing 9 healthy people does not necessarily “demonstrate” effects of haptic feedback. For AR and QoE, the paragraphs don’t justify why using or assessing these are important.

There is no particular reason to use Gait with “g” capitalized, hence we have changed it to **gait** throughout paper. This was a convention we have adopted in our research. Figure 1 was also changed to introduce valgus/varus, and not feedback.

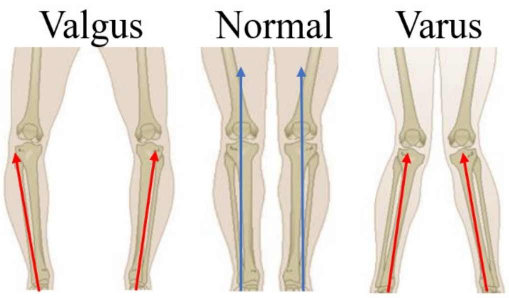

The related section was also changed to demonstrate the importance of haptic/AR feedback in current literature by also adding the following works in the manuscript:

[24] L. Turchet, P. Burelli, and S. Serafin, "Haptic Feedback for Enhancing Realism of Walking Simulations," IEEE Transactions on Haptics, vol. 6, pp. 35-45, 2013.

[25] P. Shull, K. Lurie, M. Shin, T. Besier, and M. Cutkosky, "Haptic gait retraining for knee osteoarthritis treatment," in 2010 IEEE Haptics Symposium, 2010, pp. 409-416.

[28] G. J. Diaz, M. S. Parade, S. L. Barton, and B. R. Fajen, "The pickup of visual information about size and location during approach to an obstacle," PLOS ONE, vol. 13, p. e0192044, 2018.

[29] J. Matthis, S. Barton, and B. Fajen, "The critical phase for visual control of human walking over complex terrain," Proceedings of the National Academy of Sciences, vol. 114, 07/24 2017.

[30] K. Binaee and G. J. Diaz, "Assessment of an augmented reality apparatus for the study of visually guided walking and obstacle crossing," Behavior Research Methods, vol. 51, pp. 523-531, 2019/04/01 2019.

[31] R. Kothari, K. Binaee, J. S. Matthis, R. Bailey, and G. J. Diaz, "Novel apparatus for investigation of eye movements when walking in the presence of 3D projected obstacles," presented at the Proceedings of the Ninth Biennial ACM Symposium on Eye Tracking Research & Applications, Charleston, South Carolina, 2016.

Haptic feedback was also used in enhance the realism of walking experience in multimodal environments [24], which could inform the benefit in using it to enhance user experience different applications (e.g. games, movies). Haptic feedback has also been used as an important tool in gait retraining for treatment of knee osteoarthritis [25].

The use of different AR devices was also reported for guided walking [28, 29]. These works indicate that novel AR technologies could be used in walking guidance with performance, body stability with positive impact in gaze and locomotor control [30, 31].

The text for reference [23] (now reference [26]) has been changed:

In [26], closely aligned to the focus of this work, a gait re-training system employed haptic feedback to change gait parameters including varus/valgus misalignments. The system and results served as basis for this work on informing the use of haptic feedback to capture and improve gait parameters including knee alignment.

System and Feedback Architecture

This section provides enough details about data processing. I only have some minor comments. In Figure 2, please provide photos from some other angles if the authors have some.

Figure 2 was updated and now contains photos in other angles and coordinate systems.

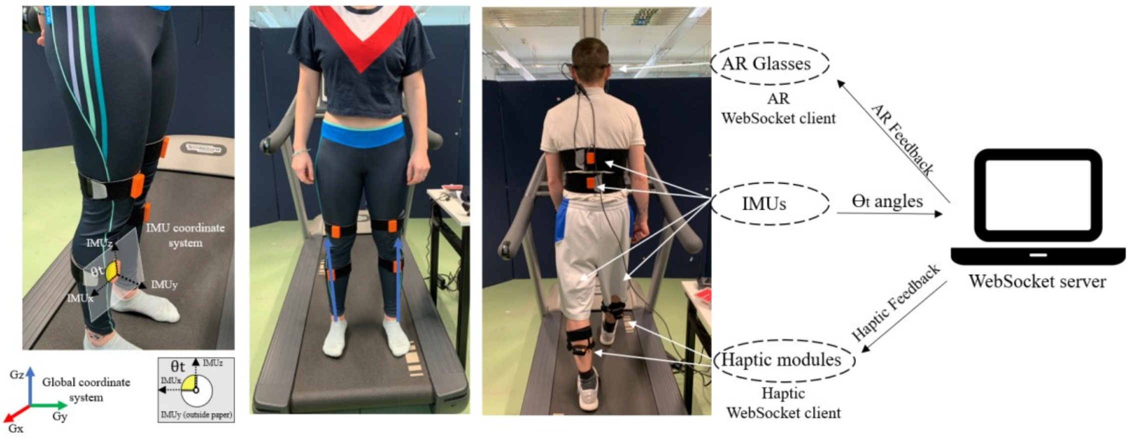

In section 3.1., did the authors use matlab and develop their own code for all calculating angles? If so, please state this somewhere. Or did the authors use software that comes with Xsens IMUs (as far as I know, Xsens provides software that calculates angles). If so, I am not sure providing all of these equations are necessary (just simply state “used

an embedded algorithm or use Xsens XXX software or something like that"). If the authors want to keep the equations, that is fine but still clarify source of data analysis.

We have changed all figures so include varus/valgus explanation, and photos from other angles. The script was developed in C#. We have only used the X-Sens Software Development Kit to allow communication between sensors. X-Sens does not provide angles for multiple IMUs. Although there are many authors doing work with angles, it is never clear how they did. All equations and algorithms are also a contribution from the author`s work (included in Supporting Information), which can help many researchers with motion capture with IMUs. These equations and script can provide angles between IMUs from all planes and joints, never stated in any other work in such detailed form.

Have the Xsens IMUs been used before for measuring valgus/varus during walking? If so, please add references so that readers know the sensors have been validated for the measurement.

Despite of the fact that X-Sens has been validated to many works in motion capture, biomechanics and rehabilitation, there is no similar work using X-Sens for varus/valgus. Some works using IMU for varus/valgus angles are in the paper as in:

[40] V. Hu, E. Charry, M. Umer, A. Ronchi, and S. Taylor, An inertial sensor system for measurements of tibia angle with applications to knee valgus/varus detection, 2014.

[41] L. S. Vargas-Valencia, A. Elias, E. Rocon, T. Bastos-Filho, and A. Frizera, "An IMU-to-Body Alignment Method Applied to Human Gait Analysis," Sensors (Basel, Switzerland), vol. 16, p. 2090, 2016.

Experimental Protocol

This section is quite poorly written, and more details are necessary for this section. Although some figures show, please described if this is a treadmill walking or something else. Also, how long (or far) was the walking? What was walking speed? Statement regarding obtaining consent form is necessary (even if it is kind of shown in a figure). If the focus is valgus/varus, why were other angles measured? How and when were the feedbacks provided?

Thanks for the suggestion. The Experimental Protocol section was updated for clarification. It was included more information about subjects, description of walking speed and consent statement. The system can measure other angles, which can lead to future work with other angles, feedback and full lower -extremity gait analysis.

IV Experimental Protocol

...After a brief explanation, written consent was obtained. Participants They were brought to the waiting room and were provided with an information sheet that fully described the experiment.

... For this experiment, we only analysed tibia angle to evaluate feedback. Full gait analysis considering all angles will be evaluated as part of a future work study.

For training and testing phases, participants were randomly assigned into two groups (Haptic/AR, and AR/Haptic) depending on which feedback the participant experienced first. Each participant in a group experienced one of the feedback

modalities and had a week break before they were presented with the alternative modality feedback. As part of the training, participants were introduced to the AR and the haptic modules as appropriate for the given test day. The devices were fitted to the participant by the principal investigator and an opportunity for adjustment was provided to ensure there was no discomfort. After sensor placement, participants were securely guided to a treadmill where they were asked to select a walking speed with which they felt comfortable (the range selected by users was between 2.5 and 4 miles per hour). Following this, in the test, the speed each participant selected was maintained for training and testing of both feedback modalities. Instructions for each feedback were explained with 3 feedback sheets (available in Supporting information) showing the difference of the three different knee states (valgus, normal, varus). Participants were aware that each leg was independent so that even though one leg was on valgus state, the other one could be aligned for example. Participants walked 2 minutes for baseline capture (no feedback), 30 seconds for feedback training, and 2 minutes (with feedback).

I think there should be another section between Experimental Protocol and Results and Analysis in order to explain statistical analysis, QoE analysis. In terms of statistical analysis, for applying for independent t-test, normality check is recommended. If variables did not pass normality test, other statistical methods are recommended.

We have explained more details about the protocol as mentioned above. Subsections for A) QoE questionnaires and B) Data processing and statistics were added into the paper. A Shapiro-Wilk test for normality was carried out before the statistical analysis and all variables came from a normal distribution ($p>0.05$).

A. QoE Questionnaire

As per [57], twelve questions asked were asked of all participants on the experience of both feedback modalities. For the subjective analysis, QoE factors were evaluated in form of questionnaires after the gait assessment phase as per Fig 6. QoE takes into consideration how system, human and contextual factors contribute to a user's perceived quality of a system [16]. The literature suggests that the accepted approach to measuring a user's perceived quality of his or her experience is based on self-reported measures via post-experience questionnaires. The developed questionnaire was used to determine an overall mean opinion score (MOS) based on feedback from users [58].

The twelve questions were developed to evaluate system utility (questions 1-3), usability (questions 4-6), interaction (questions 7-9), and immersion (questions 10-12). For each of those 4 assessment variables, 4 standard questionnaires were used as guidelines: The System Usability Scale (SUS), ITU-T methods for subjective assessment of quality, Igroup presence questionnaire (IPQ), and Computer System Utility Questionnaire (CSUQ) [52, 59-61]. The rating system used was a seven-point Likert scale to determine whether or not the participant agreed with the statement. The full questionnaire is available in [57] and per Table 3 in the results section. The ordering of the questions was randomised for the different participants to negate any ordering effects.

B. Data processing and statistics

As outlined in the methodology section, QoE and objective metrics were captured for each trial. Participants were categorized into AR and haptic. Subgroups of males (N=13) and females (N=13) were also randomly defined for gender analysis purposes. In order to compare differences across groups, a Shapiro-Wilk normality test [62] was conducted. All variables were with a normal distribution (p>0.05). A dependent samples t-test was performed on the data with 95% confidence level. For the objective analysis, we have reported differences between AR and haptic groups for number of alignments after receiving feedback, and the amount of time participants were not aligned. We have also reported the same analysis considering gender. These comparisons were done by dependent samples t-test at 95% confidence level. The QoE model (QoEMF) for each feedback for a number p of participants was designed to be average of the four-assessment metrics: Utility (UtF), Usability (UsF), Interaction (InF), and Immersion (ImF) as in (7).

Results and Analysis

Please provide some basic demographic information about the subjects. It is difficult to understand tables. For Table 1, are these number varus/valgus? So, the numbers mean the frequency of varus/valgus?

Thanks for the suggestion. Table 1 shows number of misalignments in 2 minutes' walk. Since the sample rate was high, any small misalignment was captured. We have enhanced tables explanations. There is a new discussion section that can clarify all results and tables.

Why is it important to report male vs. female? I don't think gender difference is a focus of this manuscript. Same for the other Tables.

We have reported male vs. Female as varus/valgus incidence is different for male and female for anatomic reasons [l]. Besides, if there are differences in the use case, Quality of Experience Influence Factors (IF) should be considered, hence the comparison male vs. Female.

The related work section includes now more information on the importance of reporting male and females results.

Several works have found out that valgus/varus incidence is different across gender groups [37, 38]. Anatomical differences between males and females lead to differences in knee alignment. Differences in lower extremity are potential causes of many anterior cruciate ligament in females [39, 40]. Females, in general, have wider hips than males, which is cause of many abnormalities such as knee valgus/varus, with high risk of injuries. Since the incidence of valgus/varus misalignments is different between males and females, we considered gender as an important factor to consider of this study.

There is no Discussion section?

Thanks for this good suggestion. Discussion section was added, and the authors believe tables are better explained now. We would like to thank the reviewer with all valuable suggestions.

Reviewer #2

This study targets a very important research topic that is well focused at the intersection of developing a hardware-software system and study of gait analysis-rehabilitation. The authors' major goal is to propose a system and also a series of objective and subjective metrics in order to help to improve the rehabilitation or corrections for misalignments during walking. As mentioned in the introduction this is an area that requires more research, especially when we consider the availability of state of art AR and haptic technologies. Although the goal and system implementation of this study is well defined and explained in detail, the combination of some key missing information along with lack of explanation of statistical results makes this very interesting manuscript not ready for publication at its current state. Although the paper starts very well written in the first few sections, it leaves the reader with so many critical unanswered questions toward the end. The reviewer summarizes the major concerns/questions of the work along with minor/detailed comments as follows:

Major Comments:

-The most important shortcoming of the study is where the statistical results are presented in tables 1-5.

Thanks for your time and consideration reviewing the manuscript. We have added a discussion section that discusses tables 1-4 for objective and subjective evaluations. Table 5 and old Figure 6 were removed as the authors believe it could be causing confusion in the interpretation of results.

VII Discussion

In this section we discuss the results of the comparison between AR and haptic feedback.

The results indicate that both feedback modalities reduce the occurrence of varus and valgus misalignments. AR feedback significantly reduces the number of varus misalignment (by 31%) when compared to baseline readings. Whilst the reductions for valgus (for AR) and neither varus nor valgus are significant for haptic, approximate reductions of between 9%-15% are positive. Looking deeper at the analysis, surprisingly for the male AR group, the level of reduction for varus was 45% (and 18% for valgus misalignments). Consistent with the male group, although to a lesser extent, AR feedback reduced the number of varus misalignments in 35% for the female group (not significant when compared to baseline). These results demonstrate the utility of employing both feedback, but in particular AR feedback. It also raises an interesting question to understand why females' knee did not have a significant change after receiving feedback.

For the QoE analysis, subjective evaluation of questionnaires for feedback utility, usability, interaction, and immersion was performed. Table 3 reported results of the MOS questionnaire for all participants. When participants were asked about adjustment after feedback in Question 1 ("When I received feedback, I adjusted easily and quickly."), they felt that AR was more effective in changing varus and valgus misalignments. This correlates with the objective analysis in Table 1. For the MOS questionnaire considering gender, the male group reported that they

believed their walking style changed based on the AR feedback. They also reported higher engagement when using the AR glasses than haptic devices. The female group reported higher utility of AR feedback. These difference between gender groups highlight the importance of considering human factors and employing QoE analysis in these types of novel feedback studies.

-Furthermore, there are important conclusions that are clearly inconsistent with the statistical test values reported in table 1-5.

Thanks for your suggestion. Discussion of tables to clarify results was added to the paper.

-Overall, the AR feedback condition helps improving subjects' gait characteristics only in some conditions.

The AR feedback showed significant improvement of knee alignment and the time participants were misaligned.

-Haptic feedback surprisingly deteriorates the gait characteristics in some conditions and with a low/debatable statistical significance provides improvement. This is a very important and surprising result that is not explained in the manuscript. Is this a side effect of system itself? The sensors positioning.

This result was significant only at 90% confidence ($p<0.1$). The increase of the time that participants of male group were misaligned with haptic feedback. As we kept sensor positioning consistent across all participants and trials, so the authors believe could not be from sensor positioning or a side effect of the system. Some users reported that vibration on the legs has promotes a high level of annoyance so it could possibly be the cause of this deterioration which was added to the paper.

We have also mentioned this deterioration in the results and discussion sections.

Results

Males had significant improvement in valgus time (63% $p = 0.047$). The performance for the Haptic feedback increased the number of misalignments with the male group (-49% $p = 0.06$). This suggests that the users were somewhat confused by the haptic feedback. Statistically significant difference in performance was only reported for the AR feedback in reducing varus and total misalignments with a two-tailed $p<0.05$.

Discussion

The AR group reported a MOS rating of 4.458 whereas the Haptic feedback 3.5. This result is confirmed that even not knowing performance, participants felt the AR feedback was more effective in reducing misalignments. Considering the discussion in section V.A about how participants responded to the haptic feedback (i.e. increase in misalignments), this results raises an interesting questions about the ease of understanding of haptic feedback for participants.

-As mentioned in the introduction, the goal of the study is to compare the efficiency of these two modalities. However, considering both objective and subject measures,

neither the authors nor the reader can reach a strong conclusion that which one produces a statistically significant difference? Almost all metrics show high p-values.

The aim of the paper is to compare two feedback modalities for knee alignment. When talking about rehabilitation, any significant improvement can be discussed. Findings of this study can be used for other researchers that wants to use different feedback to improve gait.

Further discussion was added in the discussion and conclusion sections.

-How many male/female subjects? It is not clear in what units the values of table 1 are presented. Second, the improvement in AR condition is different for Varus and Valgus in table 1 & 2. Why is this the case? And what should the reader learn from this difference?

We have added a section for Data Processing and Statistics section that includes more information about subjects. Table 1 reports if a group had fewer occurrences (if any) of a misalignment. Table 2 reports if a group was in time of misalignments. We have also explained Tables 1 and 2 about differences of valgus/varus for AR vs. Haptic in discussion section.

B. Data processing and statistics

As outlined in the methodology section, QoE and objective metrics were captured for each trial. Participants were categorized into AR and haptic. Subgroups of males (N=13) and females (N=13) were also randomly defined for gender analysis purposes. In order to compare differences across groups, a Shapiro-Wilk normality test [62] was conducted. All variables were with a normal distribution ($p>0.05$). A dependent samples t-test was performed on the data with 95% confidence level. For the objective analysis, we have reported differences between AR and haptic groups for number of alignments after receiving feedback, and the amount of time participants were not aligned. We have also reported the same analysis considering gender. These comparisons were done by dependent samples t-test at 95% confidence level. The QoE model (QoEMF) for each feedback for a number p of participants was designed to be average of the four-assessment metrics: Utility (UtF), Usability (UsF), Interaction (InF), and Immersion (ImF) as in (7).

-Although many parts of the experiment design, system development, hardware and software details, and coordinate transformations are explained in detail, there is some important missing information that would need some clarifications. For example, were the subjects walking at the same speed? If so, how did this not create a bias in the data collection in terms of the number of gaits for subjects with shorter gait length, given the fact that the testing duration was fixed (2 minutes)?

The experimental protocol section was updated to give a deep explanation about speed. In terms of gait cycles, the feedback was not related to the number of gait cycles, but it was constantly given to participant during 2 minutes of the test.

Please refer to the updated Experimental protocol section for clarification:

For training and testing phases, participants were randomly assigned into two groups (Haptic/AR, and AR/Haptic) depending on which feedback the participant

experienced first. Each participant in a group experienced one of the feedback modalities and had a week break before they were presented with the alternative modality feedback. As part of the training, participants were introduced to the AR and the haptic modules as appropriate for the given test day. The devices were fitted to the participant by the principal investigator and an opportunity for adjustment was provided to ensure there was no discomfort. After sensor placement, participants were securely guided to a treadmill where they were asked to select a walking speed with which they felt comfortable (the range selected by users was between 2.5 and 4 miles per hour). Following this, in the test, the speed each participant selected was maintained for training and testing of both feedback modalities. Instructions for each feedback were explained with 3 feedback sheets (available in Supporting information) showing the difference of the three different knee states (valgus, normal, varus). Participants were aware that each leg was independent so that even though one leg was on valgus state, the other one could be aligned for example. Participants walked 2 minutes for baseline capture (no feedback), 30 seconds for feedback training, and 2 minutes (with feedback).

-A simple temporal diagram seems necessary to clarify the previous question. How long was the baseline data collection? One sample trial of one subject that shows feedback/no-feedback states with theta values would be extremely helpful.

The sequence of testing was: 2 minutes baseline (without feedback), 30 seconds training (with feedback), and 2 minutes testing (with feedback). Please refer to Figure 5 and protocol information (Supporting Information) for the temporal information and full protocol we followed for each testing.

-The model is treated as a black box. Which might be the main cause of the insignificant differences in the results. How does the model transfer a difference in angle or angles into a feedback/no-feedback signal? This is not clearly explained.

We would like to thank the reviewer to point this clarification. The model constantly evaluates the current tibia angle in order to compare with threshold values. Every person has its own walking style, for this reason it is practically impossible for someone to have perfect alignment throughout every single part of the gait cycle while walking naturally. Because of that, every small change between baseline-test must be observed and tested. It is expected that as the user gets more familiar with the system and feedback, the number of misalignments could decrease even more with more testing sessions. However, an intervention study is outside of the scope of this work and can be used as future work. The text in Activation of feedback modules in section 3 of the paper was changed.

3 System and feedback Architecture

B. Feedback Module

3 Activation of feedback modules

The feedback state diagram is shown in Fig. 4. The user input is compared with the kinematic model which controls the feedback mechanism according to the activation threshold. The kinematic model was defined as per Fig. X, with activation thresholds for each feedback defined at +7o for valgus, and -7o for

varus i.e. if valgus/varus angle extended beyond the defined threshold, feedback was provided to the user. These values represent normal angle limits of knee alignment [50]. The model constantly evaluates the current tibia angle in order to compare with threshold values. Every person has its own walking style, for this reason it is practically impossible for someone to have perfect alignment throughout every single part of the gait cycle while walking naturally. Because of that, every small change between baseline-test was observed and tested.

-As a user of this system, how would one person know to correct their gait cycle based on a haptic or AR feedback? I tried to imagine this but it seems very arbitrary and unclear how would a subject correct for their gait cycle. Also, the value of 75 seconds out of 120 seconds of testing time shows 62% of the time they were not walking correctly. Is that really the case? This creates an important question about whether the feedbacks are working at all if they were still off 62% of the time. This also raises the question about the model, whether it is tunable to subjects height, wight? Or this is a general model prone to ignoring the between-subject differences?

The aim of the feedback is to correct the knee alignment for as much time as possible so that a user receives less feedback. The feedback is given constantly at frequency of 30hz, which means that any small change in alignment is detected by the system. We strictly compared the model and only considered alignment when the 2 legs are corrected, which means those 75 seconds could be from both legs. The feedback model was updated in figure 4. The model was a comparison between actual knee state and model threshold [-7,7 degrees].

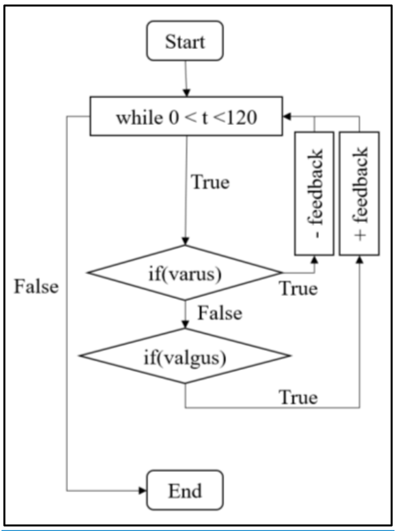

-QoE questions used in this study seems to be vague or sometimes not suitable for the task. The idea of using QoE, unfortunately, adds more confusion to the already weak statistics presented in Tables 1 & 2. For example, question #10&11 are not very clear for this study since there is not VR display involved and the subjects are of course aware of where their body is located. Even the QoE statistics doesn't help to differentiate between these two modalities of feedback based on the results in table 3,4,5.

Thanks for the suggestion. Those questions were our adaptation of standardized quality questionnaires for QoE.

[52] ITU-T, "Methods for the subjective assessment of video quality, audio quality and audiovisual quality of Internet video and distribution quality television in any environment," TELECOMMUNICATION STANDARDIZATION SECTOR OF ITU2016.

[58] A. Bangor, P. T. Kortum, and J. T. Miller, "An Empirical Evaluation of the System Usability Scale," International Journal of Human–Computer Interaction, vol. 24, pp. 574-594, 2008/07/29 2008.

[59] T. Schubert, F. Friedmann, and H. Regenbrecht, "The Experience of Presence: Factor Analytic Insights," Presence: Teleoperators and Virtual Environments, vol. 10, pp. 266-281, 2001/06/01 2001.

[60] J. Lewis and J. R, IBM Computer Usability Satisfaction Questionnaires: Psychometric Evaluation and Instructions for Use vol. 7, 1993.

We have clarified the explanation of tables 1 and 2. Question 10 “I was aware of my body whilst moving.” Dealt with proprioception that is an important variable of human motion and immersion. Question 11 dealt with immersion for focus on task. The new discussion section contains more explanation for tables 3, 4, and 5

-Why do authors think that there is an effect of gender in metrics that are not explicitly related to the perception?

The paradigm of Quality of Experience allows the researcher to evaluate different influencing factors (IF) such as system IF (e.g. AR vs VR, AR vs. haptic), user IF (e.g. age, gender), and context IF (e.g. varus and valgus). QoE studies also look at differences at perceptual level or other metrics across gender, age, level of study or previous contact with technology. More related to this study, because there are anatomical differences between males and females that affects knee alignment, we decided to also do a gender analysis. The related work section was updated to explain more why we have decided to do the gender analysis.

Several works have found out that valgus/varus incidence is different across gender groups [37, 38]. Anatomical differences between males and females lead to differences in knee alignment. Differences in lower extremity are potential causes of many anterior cruciate ligament in females [39, 40]. Females, in general, have wider hips than males, which is cause of many abnormalities such as knee valgus/varus, with high risk of injuries. Since the incidence of valgus/varus misalignments is different between males and females, we considered gender as an important factor to consider of this study.

Also, discussion section

Consistent with the male group, although to a lesser extent, AR feedback reduced the number of varus misalignments in 35% for the female group (not significant when compared to baseline). These results demonstrate the utility of employing both feedback, but in particular AR feedback. It also raises an interesting question to understand why females’ knee did not have a significant change after receiving feedback.

-Due to the mentioned major concerns, the conclusion section would need to go through a major revision along with detail answers for the mentioned questions.

The results and conclusion sections were updated to go through a deeper understanding of the study. We have included a discussion section to also explain more about results.

Conclusion

This paper presented a comparison of Haptic and Augmented Reality as feedback modalities in a gait analysis system. It compared, in terms of objective and subjective ratings, how users perceived and responded to Haptic and Augmented Reality feedback. Based on the results, the novel AR approach has significant potential as a method of gait rehabilitation. The objective evaluation tells us that AR significantly reduces the number of knee misalignment. In addition, subjective questionnaire assessment provides interesting results in terms of how users feel their walk change positively with AR feedback. The agreement of objective and subjective evaluations serves as basis of using AR as part of a rehabilitation protocol. Both gender groups considered reported that AR had greater utility than haptic feedback. The male group showed statistically significant improvement in varus, valgus, Total Misalignment, and valgus Time. Future work will further validate the assumption that AR feedback not only provides higher QoE scores but also promotes less cognitive workload in comparison with haptic as well as instantiation of the QoE model proposed above. Physiologic measures and pupillary response will also be evaluated and their inference to QoE will be analysed.

Minor Comments:

(Abstract) Shouldn't we use the term "subjective" metric? Which is well in contrast with "objective" metrics? As a suggestion, it seems the term "explicit" here is not the best choice.

Changed to **subjective** in abstract.

(Abstract) "Participant's gait improved" is a vague sentence in the abstract. Even as a general audience the immediate question is: in what way their gait was improved? Are we talking about the timing? Positioning?...

Thanks for the suggestion. We have updated the abstract to clarify improvement.

...In addition, the number of knee misalignments was reduced after users experienced AR feedback (35% improvement with AR feedback when compared to haptic).

(Line 6 & 27) Since this work is focused at the intersection of the current state of the art technologies (AR and haptics) and the well-established field of gait analysis, I strongly suggest the authors include similar studies where AR projected obstacles are implemented and the performance of the system and the human gait characteristics are reported for the purpose of fair comparison and inclusiveness. Although the references 7-10 are important in the VR/AR literature in general, however, including the contribution of the suggested references (VR/AR+gait analysis) will strengthen the argument and provides an overall picture of the field.

The pickup of visual information about size and location during approach to an obstacle GJ Diaz, MS Parade, SL Barton, BR Fajen - PloS one, 2018 The critical phase for visual control of human walking over complex terrain JS Matthis, SL Barton, BR Fajen - Proceedings of the National Academy of Sciences, 2017 Binaee, Kamran, and Gabriel J. Diaz. "Assessment of an augmented reality apparatus for the study of visually guided walking and obstacle crossing". Behavior research methods. (2018), p.1-9.

Thanks for the suggestion. We have included the works mentioned in the related works section.

Haptic feedback was also used in enhance the realism of walking experience in multimodal environments [24], which could inform the benefit in using it to enhance user experience different applications (e.g. games, movies). Haptic feedback has also been used as an important tool in gait retraining for treatment of knee osteoarthritis [25].

The use of different AR devices was also reported for guided walking [28, 29]. These works indicate that novel AR technologies could be used in walking guidance with performance, body stability with positive impact in gaze and locomotor control [30, 31].

(Line 111-112) It is more accurate to mention with minimum or low latency than zero latency, also is there a measurement over the loss of packets on the network? If not, it would be more accurate to say with minimum loss of data rather than “no data is lost”

Thanks for the suggestion. The sentence was changed to:

... IMU’s were synchronized with the computer CPU clock ensuring minimum packet loss.

(Line 135-136) Please clarify that the calibration here is referred to X-Sense IMU system calibration. Which (to my understanding) is assigning a reference coordinate system to the IMU sensors separately.

Euler angles of IMUs were calibrated separately when the user was stand. The referencing coordinate systems, in this case, is related to each IMU. In order to calculate projection angles between two IMUs, we took quaternions of each sensor into a Direction Cosine Matrix, and applied the matrix into the calibrated Euler angles (equations 1-6)

One of the figures 1,2,3,4 could be used to visualize the theta angles in order to make it easier for the reader to understand in which plane the angles are being calculated and compared with each other.

Figure 2 was updated and now it shows the plane of which the angles are being related to.

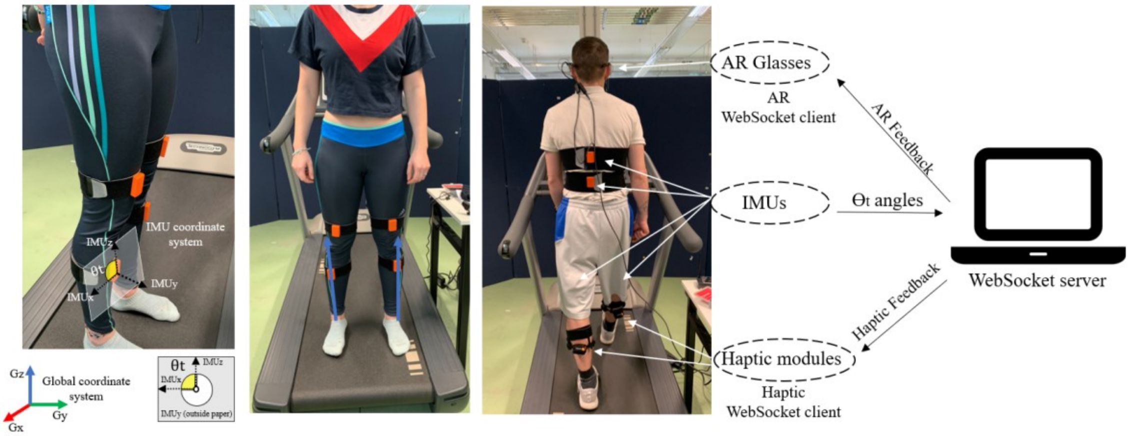

(Line 149) “is presented” ⇒ “are presented”

In this section, Haptic and Augmented Reality feedback modules are presented.

(Line 156) Were these haptic feedback modules mounted on the same position for subjects? How did you address the between-subject differences? If this was not a factor that could bias their sensation of haptic (vibration) please briefly mention why do authors think so?

We kept sensor placement consistent across all participants following same anatomical landmarks and is not a factor that could bias haptic sensation. The pre-screening assured users could properly use the AR glasses and perceive haptic sensation.

The devices were fitted to the participant by the principal investigator and an opportunity for adjustment was provided to ensure there was no discomfort.

(Line 161-166) The detailed explanation of the circuitry is highly appreciated and valuable for the purpose of reproducing the results or sharing the hardware with other researchers. However, the MOSFET is being used as the switch circuit, therefore, it seems to me the rest of the circuitry (i.e the resistor on the drain-source path) determines the current, hence the intensity of the vibration. Is that correct? If so, the sentence in line 161 seems a little misleading.

The sentence is changed to:

Vibration units are enclosed within the plastic casing. The design of the circuit contains MOSFET transistors that operate as switches. There was also a pulse width modulation control to allow precise change of the intensity of the vibration unit if required

(Line 170) in order to be consistent with AR/VR terminology instead of using “Virtual element” please use “Virtual content” (as a suggestion).

Thanks for this suggestion. Sentence changed to:

AR is a technology that allows the combination and insertion of virtual contents in the real environment.

(Line 174) Users “Field of View” FOV is also a key factor for an immersive VR/AR experience.

Thanks for the suggestion. I will include the information in the manuscript.

AR glasses are a type of wearable device that are worn like regular glasses and merge virtual and physical information in a user's field of view, which is also a key factor in immersive applications.

(Line 184) How is the kinematic model generated? Is this a general model or it is modified per subject height, weight, etc.? Please clarify.

The model is the same across subjects. The explanation of the model was clarified in figure 4.

(Line 185) Is the threshold value of +-7 in the units of angle? It seems that there's this hysteresis range between [-7,7] where the implemented algorithm considers the gait to be normal, hence no correction feedback is generated. Right? Please clarify. If so, would it be useful for the reader to know about the effect of this threshold value? What if, for one subject a threshold of 7 is perfectly fine (in terms of healthy gait characteristics) and for another user threshold value of 4? Although the authors cite a study in this field that addresses the variability, it would be helpful to briefly explain why these threshold values are selected.

The threshold was defined as an evaluation of current tibia angles to threshold values. User`s input was compared with the kinematic model which controls the feedback mechanism according to the activation threshold. Every person has its own walking style, for this reason it is practically impossible for someone to have perfect alignment throughout every single part of the gait cycle walking naturally.

The model constantly evaluates the current tibia angle in order to compare with threshold values. Every person has its own walking style, for this reason it is difficult for a participant to have perfect alignment throughout every single part of the gait cycle while walking naturally. Because of that, every small change between baseline-test was observed and tested.

As this was not a clinical study, this threshold was the mechanism we defined that every participant receives feedback even with a perfect knee alignment (anatomically speaking). Those results can inform training protocols for other researchers and is basis of refined feedback modalities (e.g. machine learning which is outside the scope of this study). It is expected that for those studies, as the user gets more familiar with the system and feedback, the number of misalignments could decrease even more with more after multiple measures. However, an intervention study is outside of the scope of this work and can be used as future work.

One major question/concern regarding the visual presentation of the circles: A very basic and important characteristic of an AR display is to avoid what is referred to as "binocular rivalry" which is presenting two conflicting visual scene to the eyes. This makes the subject experience very negative and causes severe visual discomfort and eye fatigue. Therefore, the diagrams shown in figure 4 are misleading. Is the user seeing 6 total circles? If so this diagram needs to be modified, because it shows that the right and left eye displays are shown conflicting imagery.

Thanks for the suggestion and the concern about binocular rivalry. We did not have any participant that experienced discomfort or eye fatigue with the system as our protocol

addresses. The diagram in figure 4 is right as user sees only a projection of 6 circles (3 each leg) However, the diagram in figure 4 was updated to include a better explanation of the kinematic model.

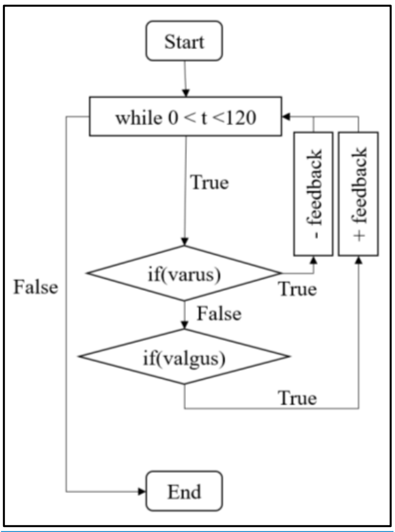

Also, in which portion of the field of view these circles are being rendered? What is their size in the visual angle? These are important questions and it will directly affect the users' perceptual experience as mentioned as one of the major goals of the study.

The rendered screen projected distance of the AR feedback was approximately 23 degrees (diagonally).

(Line 203) what is the standard deviation or any measure of variability around mean?

Average age 27.54, standard deviation of 6.27 years.

A convenience sampling approach was employed to recruit twenty-six participants (13 males, 13 females) with an average age of 27.54 (± 6.57) years

(Figure 5 Caption) "consistent"

Typo fixed.

(Line 217) Location on which parts of the body?

On each leg for both haptic devices.

How long was the training testing session? How fast was the speed of testing/training sessions compared to baseline? How did the baseline data were used?

As mentioned, the Experimental Protocol section was updated to include clarify information. The full protocol is available in the Supporting Information. After sensor placement, participants were securely guided to a treadmill where they were asked to select a walking speed that they felt comfortable walking (between 2.5 and 4 miles per hour). The selected speed for each participant was kept for training and testing for both days the participant was tested. The baseline was used to compare misalignments with and without feedback (baseline vs. tests). We have also captured physiological metrics (blood volume pulse, heart rate, electrodermal activity, and eye tracking) during baseline and tests. These metrics will be used for next publications.

(Line 237) Response time of feedback with respect to what? Is this a duration? A very important part of the manuscript, yet after reading this section multiple times, I'm not still sure what these metrics are? The authors are encouraged to use a very simple temporal diagram and mark significant events (states) for a sample trial for one subject. This visualization along with a modified explanation seems to be necessary.

We have clarified the sentence in manuscript

We report, for each state: the time users needed feedback and the number of times the participant needed feedback (feedback cue) for the same time interval (2 minutes).

The experimental protocol section was updated to clarify the steps of each trial. There is a file in supporting information that shows the detailed protocol.

What is the "same time interval"? 2 minutes? Please clarify.

Same interval refers to 2 minutes.

Is 2 minutes of data enough to conclude? This is more of a general question rather than concern. Please explain to a more general audience why this provides enough sample for a walking study. Especially, it is not clear how long the feedbacks last on the haptic device or on the display? Do feedbacks immediately disappear as soon as the system reports smaller misalignments relative to the threshold? Or there is a fixed feedback duration time?

In order to keep trials with same time, we decided to capture feedback data with 2 minutes. As this was not an interventive study, 2 minutes were enough to compare with 2 minute baseline. Explanation of different feedback was updated in system architecture section under Activation feedback subsection.

The feedback in the Haptic module was presented as vibrations on each leg whenever the participant's tibial angle was above or below the activation thresholds for valgus and varus. The correct alignment of each leg resulted in "no vibration" (i.e. no feedback provided) on the Haptic bracelet. The objective given to the participant was to receive the least amount of vibration as possible. This was explained during the training phase in section IV. Participant were told that no feedback from haptic means they are in correct alignment. The feedback in the AR module was presented as circle visualizations on the AR glasses (see Fig. 4 and Fig. 5). Again, whenever the tibial angle was above or below the activation thresholds for valgus and varus. For each leg, three circles control the states of the knee according to valgus and varus angles. The correct alignment of each leg is achieved when the blue circle in the middle is lit. The objective given to the participant is to keep the circles blue during trial. For AR module, user sees a projection of 6 circles in their field of view (3 of each leg as per Figure 5)

Experimental protocol

Instructions for each feedback were explained with 3 feedback sheets (available in Supporting information) showing the difference of the three different knee states (valgus, normal, varus). Participants were aware that each leg was independent so that even though one leg was on valgus state, the other one could

be aligned for example. Participants walked 2 minutes for baseline capture (no feedback), 30 seconds for feedback training, and 2 minutes (with feedback).

A follow-up question/concern: One could argue that the total misalignment might not be the best metric to compare. Because a person with shorter gait, during 2 minutes of walking would provide larger number of gaits compared to someone with a much larger gait length (which is obviously correlated to height). In many similar studies, the adjust/unbias the metric relative to the gait length or gate cycle.

The metric is temporal (2 minutes) rather than the number of gait cycle for example. With this design, the individual anthropometrics of participants (e.g. leg length) could be unconsidered.

(Line 257) “combination”? ⇒ “Average”

Changed it to **average**

An important question: For both tables 1 and 2, are the reported values averaged over subjects? If so, please also consider reporting the variation from the mean. Table1: In which physical unit the values are represented? Is this the total angle? Or is this in time? Please mention the number (N) of male and female participants from which the table 1 is generated. Although p-value is an important measure of statistical significance, however, without knowing the effect size the interpretation of the results will be incomplete. There are many ways to calculate this as it is implemented in almost all statistical analysis tools. (https://www.ncbi.nlm.nih.gov/pmc/articles/PMC3444174/)

Thanks for this suggestion. The number of males and females are reported in the paper. The table 1 contains the number of misalignments for baseline and test and not the angles or variation of angles. In many studies reporting this variation, effect size could be a good strategy to look at variances and where they come from. We discussed results for p values as t-tests were more appropriate for all results (table 1-4). Thanks for this excellent suggestion. We will report the interaction size for those metrics and will consider for other implementation and design.

Comparing Tables 1 & 2: why do we see more improvement in Varus (all subjects) in table 1 but more improvement in Valgus in table 2? These are the same data from the same subjects, one is represented in the unit of time (table 2) and the other one in the units of degree (I think). This seems to be an inconsistency, right?

These are addressed in the discussion section now. Table 1 refers to number of feedbacks in 2 minutes and table 2 refers to the time users needed feedback to correct alignment. We have clarified this matter.

In table 2: the negative values show that the feedback mechanism for haptic conditions made the subjects confused or unclear about how they are supposed to correct for their gait. Could this be attributed to the implementation, circuit positioning or there is a perceptual justification for it?

This result was significant only at 90% confidence ($p<0.1$). The increase of the time that participants of male group were misaligned with haptic feedback. As we kept sensor positioning consistent across all participants and trials, so the authors believe could not be from sensor positioning or a side effect of the system. Some users reported that

vibration on the legs has promotes a high level of annoyance so it could possibly be the cause of this deterioration which was added to the paper.

We have also mentioned this deterioration in the results and discussion sections.

Results

Males had significant improvement in valgus time (63% p = 0.047). The performance for the Haptic feedback increased the number of misalignments with the male group (-49% p = 0.06). This suggests that the users were somewhat confused by the haptic feedback. Statistically significant difference in performance was only reported for the AR feedback in reducing varus and total misalignments with a two-tailed p<0.05.

Discussion

The AR group reported a MOS rating of 4.458 whereas the Haptic feedback 3.5. This result is confirmed that even not knowing performance, participants felt the AR feedback was more effective in reducing misalignments. Considering the discussion in section V.A about how participants responded to the haptic feedback (i.e. increase in misalignments), this results raises an interesting questions about the ease of understanding of haptic feedback for participants.

(Line 294) “group”

Typo corrected.

The choice of questions for this study could be revised. For example, question #10 and #11 is mostly used for a VR experiment in order to check whether the subject would sense a virtual representation of their body parts, such as hand, feet, etc. Therefore, there is no surprise that the two modes of feedback are not perceived differently from the subjects’ point of view.

Thanks for this suggestion. We have a new subsection dedicated to explain the questionnaire under experimental protocol section.

Those questions were our adaptation of standardized quality questionnaires for QoE.

[52] ITU-T, "Methods for the subjective assessment of video quality, audio quality and audiovisual quality of Internet video and distribution quality television in any environment," TELECOMMUNICATION STANDARDIZATION SECTOR OF ITU2016.

[58] A. Bangor, P. T. Kortum, and J. T. Miller, "An Empirical Evaluation of the System Usability Scale," International Journal of Human–Computer Interaction, vol. 24, pp. 574-594, 2008/07/29 2008.

[59] T. Schubert, F. Friedmann, and H. Regenbrecht, "The Experience of Presence: Factor Analytic Insights," Presence: Teleoperators and Virtual Environments, vol. 10, pp. 266-281, 2001/06/01 2001.

[60] J. Lewis and J. R, IBM Computer Usability Satisfaction Questionnaires: Psychometric Evaluation and Instructions for Use vol. 7, 1993.

We have clarified the explanation of tables 1 and 2. Question 10 “I was aware of my body whilst moving.” Dealt with proprioception that is an important variable of human motion

and immersion. Question 11 dealt with immersion for focus on task. The new discussion section contains more explanation for tables 3, 4.

Question #9 Consistent feedback with respect to what? This is a very vague question unless the experimenter has provided additional information to the subject not mentioned in the manuscript. Please clarify.

We have explained the full questionnaire to participants after testing. Consistent feedback as a metric of que flow of feedback throughout the testing.

(Line 298-299) The statistical significance test presented in the table clearly disputes this sentence. The correct conclusion here so far is: the statistical significance test reveals no difference between AR and haptic feedback based on the QoE metric. Except question 1, which could also be ruled out since there is only 1 out of 11. Unfortunately, this is the weakest part of the paper that requires a clear explanation along with the authors' intuition.

For any QoE study, we look at indicatives using different metrics (e.g. performance, questionnaires, and physiological measures). Those questions inform how users perceive and interact with the system; therefore, they are valuable contribution. We advise the reviewer to kindly look at the new discussion section where the authors clarified results and discussion of questionnaires.

(Line 306) Please avoid using subjective evaluations of the results such as "interesting" as the reader might find these differences very confusing rather than interesting. For example, why do gender matter in #2 which refers to the change in walking? As stated in the table this metric is a utility feature of the system and has very little to do with perceptual judgments (for example being involved with system #12). This seems to be an unanswered question in this study. (Line 307-313) Please refer to the same comment for lines 298-299. There's no statistical significance (except a few questions with unclear gender differences).

The discussion section was updated, and it clearly explains tables and results.

There are some spaces every few lines that separate the letters of a single word (i.e. lines 230 "discussion").

We have proofread the paper and corrected those empty spaces. We would like to thank the reviewer with all valuable suggestions.
