# Supplementary material for: A Quality of Experience assessment of haptic and augmented reality feedback modalities in a gait analysis system
Source: PLoS One. 2020 Mar 23;15(3):e0230570. doi: 10.1371/journal.pone.0230570 (PMC7089541; doi:10.1371/journal.pone.0230570)
Supplement: Attachment — Submitted filename: Rebuttals PlosOne4 NM OC.DOCX [file pone.0230570.s003.DOCX]

**A quality of experience assessment of haptic and augmented reality feedback modalities in a gait analysis system**

Thiago Braga Rodrigues, Ciarán Ó Catháin, Noel O’Connor, Niall Murray

This document includes the reviewers’ comments (black text), responses to the reviewer’s comments (in blue) and the content which was added to the revised version of the paper (**in blue bold**). We wish to thank all reviewers for the time and efforts they put into reviewing this paper. Their constructive comments and critique have greatly improved the version under review now and we genuinely thank them for this.

Reviewer(s)' Comments to Author:

**Reviewer #1**

Thank you for addressing my comments in this version. I do have some more comments that need to be addressed. In the introduction, it is still not very clear why AR feedback and Haptic feedback need to be studied. What are the limitations of current feedback tools (2D screens; haptic; audio; expert guidance), and how the current tool that the authors tested those limitations? This needs to be cleared up to justify the need of this study.

Thank you again for your constructive comments and highlighting the requirement for us to justify the approach. The existing literature, as the reviewer correctly points out, presents various feedback mechanisms are widely used in many research (discussed in the related work). However, novel immersive technologies such as Augmented Reality (AR) are emerging that potentially have utility. AR is relatively speaking unexplored in terms of gait feedback and in this work we wanted to evaluate how users perceived the feedback and the utility of AR compared to the state of the art Haptic feedback approach. In addition, current feedback technologies have some limitation:

- 2D screens – This approach does not allow the user to walk freely in any direction they want, they always need to walk towards the screen and must have their head up facing the screen.
- Audio – In order to comply to the audio guidance, users need to clearly understand the guidance which can be confusing. The existing literature also highlights that visual based feedback has higher utility than audio for gait re-education (ref [26] paper).
- Expert guidance – Of course this has many benefits, but requires the user to attend an expert clinic, the expert to be available and additional costs for always paying an expert, and it is based on subjectivity of the clinician.

These current tools have been shown to have utility but as discussed above and in the updated version of the paper, they also have individual limitations. We have updated the paper to highlight these limitations. The paper also provides information via objective analysis (e.g. improvement after AR feedback) and subjective analysis such as questionnaires to demonstrate the utility of using AR. AR is shown to be portable, wearable and immerses the user. Hence, to justify the needs for the study, the updated paper contains limitations of existing approaches and highlights the potential of the novel AR based approaches.

We have updated the introduction to include this information and further clarification on justification of this study.

**Feedback is a powerful tool for motor skill learning and helps with the sensory perceptual information as part of performing and learning a skill [6].** **The accuracy of exercise performance with feedback in physiotherapy influences the healing process of the patient greatly. Crucial to successful rehabilitation is for the patient to understand the feedback, be it from a clinician or system. Many rehabilitation and retraining systems for physiotherapy and exercise learning use feedback as an important tool for patient learning and progress [7]. Some of the feedback modalities in training systems include: 2D screens; haptic; audio; expert guidance; and in more recent times Virtual Reality (VR) and Augmented Reality (AR) [8-10].**

**All of the** **different feedback approaches have advantages and disadvantages. For example, with 2D screen feedback, the user is limited in terms of the direction they can walk i.e. they are always required to walk towards the screen and must have their head up facing the screen. Audio guidance addresses this issue, but users need to clearly understand the guidance. With audio, this has shown to be an issue and a source of confusion [8]. The use of expert guidance has many benefits, but it requires the user to attend an expert clinic; the expert to be available; and is based on subjectivity of the clinician. Considering the current feedback modalities, 2D screens, audio, expert guidance, haptic has been shown to support the greatest user improvement for gait re-training and feedback [11,12]. However, this requires the accurate placement of the haptic feedback displays. AR has been very successful in education and considering the limitations of existing approaches, and the potential for AR as a portable, wearable and visual feedback modality, there are opportunities in applying immersive technologies as a feedback modality.**

**AR is an interactive experience in a real-world environment whereby real-world objects are augmented with virtual information [13].** **The future of AR points to a deeper use of technology** **augmenting human performance across a range of application domains [14].** **Despite the countless possible applications and advances in the industrial sector, the understanding of user perception of AR technology is limited. Hence, there is a real need for user studies to determine the usability and utility of these technologies. This can be addressed through the Quality of Experience (QoE) framework.** **The use of AR via wearable smart glasses in the field of gait rehabilitation is certainly an area under researched to-date. This study investigates if AR has the potential to be a light weight and portable feedback alternative for rehabilitation protocols considering both performance and subjective user experience evaluations through a QoE evaluation.**

In terms of the reviewer comments “and how the current tool that the authors addressed those limitations?”,

to address this valid query, we have used objective (e.g. number of misalignments after feedback) and subjective evaluation of questionnaires to test system utility (e.g. “When I received feedback, I adjusted easily and quickly.”), usability (e.g. “The feedback was easy to understand.”), immersion (e.g. “I was aware of my body whilst moving.”). We have concluded that AR is portable, wearable and immerses the user. This is the aim of the QoE part of the paper.

**...to test some of the shortcomings mentioned above, in our system, we have provided a questionnaire aiming to understand how users perceived utility, usability, interaction, and immersion of the proposed AR system. In terms of system utility (e.g. easiness to adjust to feedback), usability (e.g. feedback easy to understand), interaction (e.g. how users interact with feedback), immersion (e.g. awareness of body whilst moving.)**

In the Methods, so the authors used their own code to calculate the knee angles? Has the code been validated somehow (Even in small sample)?

We designed and implemented our own algorithm and code to calculate angles. This code was validated with the gold standard VICON system in our previous published works (reference **[43]** paper)**.** These validations compared several motion capture systems with the Inertial sensor-based Motion Capture for hip and knee angles (“An evaluation of a 3D multimodal marker-less motion analysis system”, published at ***ACM*** [***MMSys19***](https://dl.acm.org/citation.cfm?id=3304109.3306236)), and we also compared our system with VICON with bootstrap angle analysis and spatiotemporal gait variables (reference **[44]** paper). This second study was published in the journal [***Multimedia Tools and Application***](https://link.springer.com/article/10.1007/s11042-019-08275-9). As result of these previously published works, we validated our approach, algorithm and compared our system and code for accuracy against the gold standard VICON.

All equations and algorithms are also a contribution from the author`s work (included in Supporting Information), which can help many in the field to replicate these equations. That is why it is important to publish this work in an open-access journal such as Plos One.

**The methodology described above was part of the contribution from our previous works. In those works, a full validation of the use of IMUs for motion capture and gait analysis was performed against the gold standard VICON system. Further details on the multi-IMU angle conversion and streaming protocol is available in [44, 45] for the interested reader. We have also made available the code in the Supporting Information.**

Discussion needs to be much more deeper. The authors did not really discuss what is expected to be. For example, are the authors' findings expected? Were they in line with previous reports? If so, what does that mean? If not, why were the results different? Also, how and why should we care about these results? AT LEAST, questions above need to be discussed.

We have addressed these comments in the updated discussion section. Here we provide direct answers for each of the points from the reviewer:

Hypothesis and findings.

Our hypothesis considering state of the art research was that the haptic feedback would yield the best results. This assumption was based on existing studies and primarily because the “haptic” feedback devices are “placed” close to where the changes in feedback are required.

Relationship with previous reports

As reported, the results were different from what we expected. Surprisingly, the AR feedback could not only reduce the number of misalignments, but subjective questionnaire analysis demonstrated that users reported that AR feedback was better than haptic to reduce the number of misalignments.

Why should we care about these results?

Feedback and users’ responses to same is an important topic to understand. In our use case, it can have a significant impact on a person’s Quality of Life. Reducing misalignments can also reduce the injury incidence more. Considering that many existing research works have been conducted using current feedback tools such as 2D screen and haptic, this study can be a new paradigm in using immersive technologies in gait re-training and promotion of rehabilitation protocols.

These results are important for the research community and was also a good indicator that we are going on the right path for future work.

We have updated the discussion section with further clarification of results

Discussion

**In this section we discuss the results of the comparison between AR and haptic feedback. Due to the fact that haptic feedback has been reported as a viable feedback modality across many fields such as rehabilitation and gait re-education, our assumption was that haptic feedback would report better results in terms of user performance (and also possibly QoE).** **Haptic information is given directly at the joint that the user needs to change whilst AR feedback the participant needed to process visual information and change the leg related to that change. Surprisingly as seen in the results, AR feedback not only reduced the number of misalignments, but from the subjective questionnaire analysis, users reported that AR feedback helped to reduce the number of misalignments better than haptic.**

**...Feedback and users’ responses to same is an important topic to understand. In our use case, it can have a significant impact on a person’s Quality of Life. Reducing misalignments can also reduce the injury incidence more. These results are important for the research community and was also a good indicator for future work, where we will extend the research for understanding physiological measures and what happens in a clinical setup for males and females**

**....Considering that many research works were conducted using current feedback tools such as 2D screen and haptic, this study can be a new paradigm in using immersive technologies in gait re-training and promotion of rehabilitation protocols.**

All changes in introduction and discussion are in the updated manuscript. We would like to thank the reviewer again with all valuable suggestions.

**Reviewer #2:**

Thanks to the extensive edits and modifications made by the authors, the core arguments of the paper, the explanations required for an easier understanding of the goals, the analysis and interpretation of the statistical results are significantly improved.

The major questions regarding the manuscript are either addressed in the updated version or explained in the response document.

Going through the revised manuscript there are no major edits seem to be required at its current stage. The efforts put behind revising the manuscript is highly appreciated and it clearly manifests itself in the outcome.

The only minor reminder would be making sure that the quality of figures 5,6,7 are high enough so that after compression it would still look decent.

Good luck with your future research endeavors.

Thanks

We would like to thank the reviewer with all valuable contributions. We will make sure that figures look decent after compression.
